# Linking Protein Motion to Enzyme Catalysis

**DOI:** 10.3390/molecules20011192

**Published:** 2015-01-13

**Authors:** Priyanka Singh, Thelma Abeysinghe, Amnon Kohen

**Affiliations:** Department of Chemistry, University of Iowa, Iowa City, IA 52242, USA; E-Mails: priyankanarendra-singh@uiowa.edu (P.S.); donthelma-abeysinghe@uiowa.edu (T.A.)

**Keywords:** dihydrofolate reductase, thymidylate synthase, kinetic isotope effect, protein motions, dynamics

## Abstract

Enzyme motions on a broad range of time scales can play an important role in various intra- and intermolecular events, including substrate binding, catalysis of the chemical conversion, and product release. The relationship between protein motions and catalytic activity is of contemporary interest in enzymology. To understand the factors influencing the rates of enzyme-catalyzed reactions, the dynamics of the protein-solvent-ligand complex must be considered. The current review presents two case studies of enzymes—dihydrofolate reductase (DHFR) and thymidylate synthase (TSase)—and discusses the role of protein motions in their catalyzed reactions. Specifically, we will discuss the utility of kinetic isotope effects (KIEs) and their temperature dependence as tools in probing such phenomena.

## 1. Introduction

Enzymes are involved in a well-orchestrated series of metabolic reactions that facilitate many processes essential to life. These biocatalysts greatly accelerate these chemical reactions, achieving rate enhancements as high as 10^25^ relative to the corresponding uncatalyzed reactions [1]. An understanding of what makes enzymes so efficient is thus of great intellectual and practical interest, as it can lead to both rational drug design and strategies for the design of biomimetic catalysts. Over the past several decades, many sophisticated techniques have been used to investigate the mechanisms that allow catalytic activity, ranging from simple assays to high-resolution X-ray crystallography and spectroscopy. These studies have offered insights about many structural and conformational characteristics necessary for understanding the catalytic activity of enzymes [2,3].

Despite numerous theories that have been proposed during the past century—from Fischer’s “lock and key” model [4] to Pauling’s and Koshland’s induced fit theory [5,6]—debate continues about the origin of the catalytic power of enzymes [5,7]. There is still a need for new ways of addressing pressing questions that current theories of enzyme catalysis and dynamics have not resolved. In particular, the participation of residues remote from the active site in the catalyzed bond activation is of great interest, as is the long-range communication of residues across the enzyme. Better understanding of global effects across the protein may reveal the purpose and function of the enzyme structure beyond the active site. While the widespread availability of rigid crystal structures has revolutionized the field of structural biology, a more dynamic view of enzyme catalysis, based on advances in both computational and experimental enzymology, holds appeal across scientific disciplines. Since the term “dynamics” could have different interpretations in different contexts and by different researchers, we should first define its use in this review. Dynamics here address any motion of the atoms that compose the system under study: reactants, protein, and solvent. Dynamics that are associated with the catalyzed reaction and its rate, are those that bring the reactants-state to the transition state (TS, or the tunneling-ready state, TRS, if nuclear QM tunneling is part of the barrier crossing process, *i.e.*, QM delocalized TS), dynamics associated with barrier re-crossing events, and those that dissipate the TS toward product. Those motions are assumed to be in thermal equilibrium with their environment, and this review does not invoke any non-equilibrium, non-statistical, or otherwise non- non-Boltzmann distribution of states. Of course this assumption is broken when the event in question is faster than the thermal/vibrational dissipation of the heat, but this is not at the focus of this review, and not needed to explain the phenomena described below. This topic and a more rigorous explanation of how dynamics are incorporated into transition-state theory (TST) or Marcus-like theories has been recently reviewed elsewhere [8], for the benefit of interested readers. Here we focus on the applications and incorporations of different factors that affect enzyme catalysis.

It has become apparent that in order to understand the factors that influence the rates of enzyme-catalyzed reactions, the dynamics of the protein, as well as its environment, must be considered. Enzyme motions on a broad range of time scales play important roles in various aspects of function, including substrates’ binding, the chemical conversion of substrate to reactants, and product release [7]. It has been proposed that motions on different time scales, of both residues in and far from the active site of the protein, participate in catalysis. Identifying and characterizing these motions is a challenging task (especially when considering distal residues, as it is difficult to predict their function and effect on the active site based on X-ray crystal structures alone). However, numerous enzymatic studies of model systems—including dihydrofolate reductase, thymidylate synthase, human purine nucleoside phosphorylase, and soybean lipoxygenase-1—suggest that mutations distant from the active site affect chemical bond activations in that site, hence, supporting long-range protein motions [9,10,11,12,13,14].

The current review describes selected experimental approaches that explore the relationship between protein dynamics and the bond activations of two enzymes, dihydrofolate reductase (DHFR) and thymidylate synthase (TSase). First, we describe the global network of coupled motions in DHFR, and studies of an isotopically substituted enzyme (item 2). Next we present studies of TSase and the relationship between its motions and the chemical conversions it catalyzes (item 3).

## 2. Dihydrofolate Reductase (DHFR)

### 2.1. Introduction

**Figure 1:**
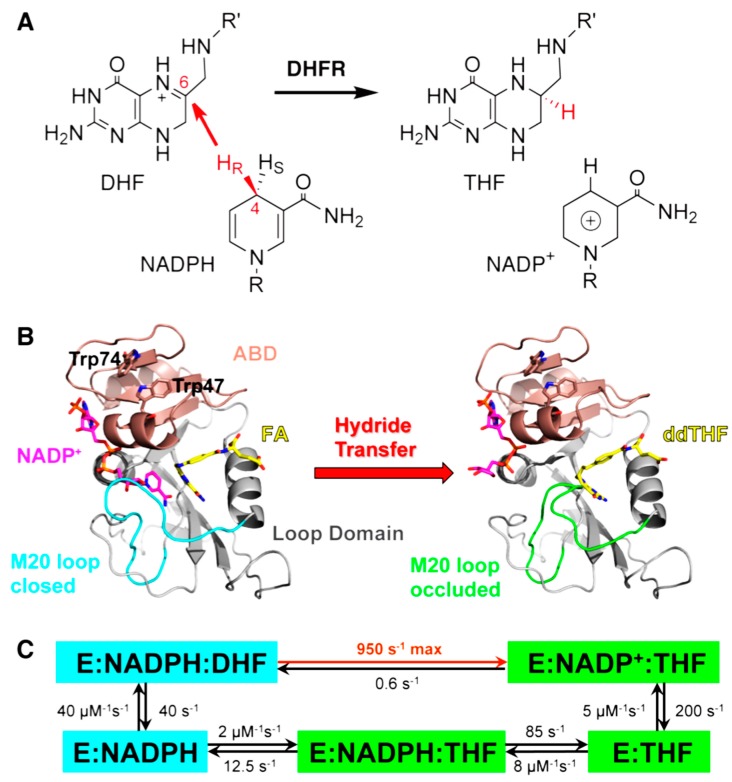
(**A**) DHFR catalyzes the stereospecific transfer of the pro-*R* hydride of C4 on NADPH to C6 of protonated N5-DHF, producing THF and the oxidized cofactor NADP^+^. (**B**) The active site cleft of DHFR divides the protein into two domains: the adenosine binding domain (ABD, residues 38–88) binds the adenosine moiety of the cofactor NADPH, while the loop domain (~100 residues) is dominated by three loops surrounding the active site. The ternary complex of DHFR with NADP^+^ (magenta) and folic acid (FA, yellow) mimics the Michaelis complex (structure on the left, PDB code 1RX2). The M20 loop (cyan) closes over the active site to ensure close proximity of the hydride donor (C4 of NADPH) and acceptor (C6 of DHF). The ternary complex of DHFR with NADP^+^ (magenta) and 5,10-dideazatetrahydrofolic acid (ddTHF, yellow) mimics the product complex (structure on the right, PDB code: 1RX4): here the M20 loop (green) protrudes into the binding site of the nicotinamide ribose moiety of the cofactor to facilitate product release. (**C**) Under cellular conditions of *E. coli* (with abundant NADPH concentrations), DHFR cycles through 5 kinetic intermediates, which are colored according to their M20 loop conformations (cyan: closed; green: occluded). The rate constants of each step are from [18]. The maximum (pH-independent) hydride transfer rate (950 s^−1^) was obtained from non-linear regression of the pH dependence of observed rate constants (pH 5.5–9.0) in stopped-flow experiments [18]. Reprinted from [19] with permission from the American Chemical Society.

DHFR (E.C. 1.5.1.3) is a vital enzyme in folic acid metabolism and in the synthesis of genetic material in many organisms [15]. DHFR from *Escherichia coli* (*E. coli* or *ec*) is a small (18 kDa), NADPH-dependent oxidoreductase that catalyzes a N=C double bond reduction via protonation of the nitrogen and a hydride transfer from the nicotinamide cofactor to the carbon. DHFR converts 7,8-dihydrofolate (H_2_F or DHF) to *S*-5,6,7,8-tetrahydrofolate (H_4_F or THF), using NADPH as a reducing agent (Figure 1A) [16,17,18]. The structure and kinetic mechanism of DHFR has been extensively characterized, making it a model enzymatic system in numerous experimental and theoretical studies [5,7,18,19,20,21,22,23,24,25,26,27]. The DHFR protein fold contains large loop regions connecting four flanking *α*-helices and a central eight-stranded *β*-sheet (Figure 1B). DHFR undergoes large-scale backbone motions during the catalytic cycle; its M20 loop fluctuates between closed and occluded conformations in conjunction with binding and release of the substrates and products (Figure 1C) [17,28,29].

DHFR from *E. coli* has been extensively characterized by a range of biophysical techniques. Both crystallographic and NMR studies show that the M20 loop region of this enzyme adopts several conformations relative to the active site as the catalytic cycle progresses, and suggests that the movement of this loop might modulate the turnover rate by limiting the rate of product dissociation [17,27,30,31]. In another study, fluorescence microscopy and ensemble kinetics were used to study conformational transitions associated with enzyme catalysis [32]. Recently, Hecht, Benkovic and co-workers introduced two pyrenylalanine chromophores into DHFR, which led to excimer formation at the reactive state [33]. This experiment provided a more direct demonstration that *k*_cat_ is governed by a conformational change *after* the hydride transfer step. Another NMR study of DHFR in complex with a variety of ligands also suggested that changes in the dynamics of the enzyme may be correlated with kinetic events along the catalytic cycle [34].

The notion that the dynamic behavior of remote residues might influence events at the active site has been argued in the case of several enzymes [13,35,36,37,38]. DHFR served as one of the better-studied systems in the context of a global dynamic network associated with catalyzing a chemical conversion at its active site. Along with experimental studies, theoretical investigations utilizing molecular dynamics (MD) and quantum mechanical/ molecular mechanical (QM/MM) simulations (as well as bioinformatic studies of genomic coupling and coevolution) suggest that enzyme dynamics play a role in catalysis, and support the presence of a global dynamic network of residues in *E. coli* DHFR [16,39,40,41,42,43]. The term “dynamic network” in this context refers to all the residues whose motion is coupled (to each other) and is part of the reaction coordinate. In addition to the reactants, in enzyme catalysis the reaction coordinate includes atoms of the solvent and the protein. While it is intuitive that such network includes residues in the enzyme’s active site, refs [16,39,40,41,42,43] suggested that several residues far from the active site are also part of that network.

Kinetic studies of a series of DHFR mutants of residues remote from the active site further suggest that long-range enzyme motions affect this enzyme’s catalyzed chemistry [42]. The data indicate that some of the remote residues behaved in a synergistic fashion (*i.e.*, two single mutants caused changes in single turnover rates whose sum was smaller in magnitude than the change generated by the corresponding double mutant); this result strengthens the case for long-range protein motions. Therefore, the complete picture that emerges from several studies is of a “global network” of residues in DHFR that are coupled to each other and correlated to its chemistry [16,40,41,43,44]. It was from this perspective that kinetic isotope effect (KIE) experiments were undertaken to further evaluate the degree, nature, and impact of the proposed dynamic network in *E. coli* DHFR.

Measurement of the temperature dependence of intrinsic KIEs is a sensitive probe of the nature of the reaction coordinate and the nature of chemical reactions [7,8]. Regardless of the details of specific models used in the data interpretation as presented in those refs, temperature independent KIEs result from a narrow distribution of DADs at the TS (TRS for QM delocalized TS), *i.e.*, high frequency of DAD fluctuations at the TS. Temperature dependent KIEs, on the other hand, indicate a broader distribution of DADs at the TS (poor reorganization of the reactants at the reactive state and lower frequency of DAD’s fluctuations). For moderate temperature dependence this distribution can still be mimicked by one frequency (harmonic oscillator). For steeper temperature dependent KIEs, the inharmonic distribution can no longer be presented by single frequency (e.g., see Warshel’s DAD-potentials in ref [22]), as discussed in more details in ref [45]. In most wild type (WT) enzymes, the intrinsic KIEs for the WT DHFR have been shown to be temperature independent [46]. According to the phenomenological models used to analyze such data [7,44,47,48,49,50,51] this suggests that the enzyme active-site dynamics bring the donor-acceptor distance (DAD) to a well-defined ensemble of short and narrowly-distributed DADs for hydride transfer [8,46]. As a benchmark for the relationship between the temperature-dependence of KIEs and DAD distribution, we use a serial mutation of an active site residue that directly affects the DAD [52,53]. Isoleucine at position 14 (I14) holds the nicotinamide ring of the cofactor (NADPH) close to the hydride acceptor (DHF) and helps in maintaining an optimal DAD. I14 was studied through a series of mutations that were chosen to decrease the side chain length without substantially perturbing active site electrostatics. Mutations from I to V, A and G were made, and the distribution of DADs was assessed by molecular dynamic simulation, indicating that as expected, DAD increased and had broader distribution as the side chains decreased in length from isoleucine to glycine. The isotope effects on the slope of the temperature dependence of the intrinsic KIEs gradually increased as the size of the amino acid at position 14 decreased. Together the findings suggested that a longer and more broadly-distributed DAD results in steeper temperature dependence of the intrinsic KIE. Furthermore, the studies of the active site residue I14 serve as a model for understanding the role of distal residues whose functions in catalysis are not easy to understand structurally [52,53].

### 2.2. Effect of Remote Residues on the DHFR Catalyzed Chemistry

**Figure 2:**
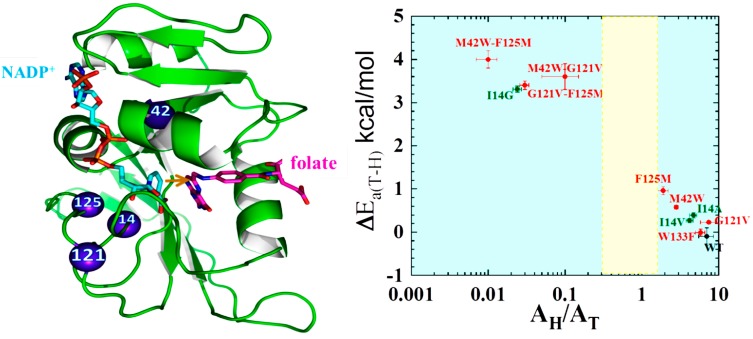
Roles of active site and distal residues on the DHFR catalyzed reaction. Left panel: Structure of WT-DHFR (PDB Code 1RX2), with folate in magenta and NADP^+^ in light blue. A yellow arrow marks the hydride’s path from C4 of the nicotinamide to C6 of the folate, and the residues studied in ref [10] are marked as blue spheres. Right panel: Presentation of the temperature-dependence parameters of intrinsic KIE for WT (black), distal (red), and active site I14 (green) mutants of DHFR, where error bars represent standard deviation. The ordinate is the isotope effect on the activation energy (ΔE_a_) and the abscissa is the isotope effect on the Arrhenius pre-exponential factor (A_H_/A_T_) both of which were determined from a non-linear regression of the intrinsic KIEs to the Arrhenius equation. The yellow block represents the semi-classical range of the Arrhenius pre-exponential factor (0.3–1.7) [49]. Reproduced from ref. [10] with permission from the American Chemical Society.

Figure 2 focuses on studies of temperature dependence of the intrinsic KIEs with remote single mutants M42W, G121V, F125M, W133F and their double mutants. Three predictors guided these studies: (i) Bioinformatic analysis implicated residues M42, G121, and W133 as “strongly coupled”, whereas F125 was classified as only weakly coupled [16]; (ii) QM/MM calculations specifically examined motion of the protein along the reaction coordinate, and M42, G121 and F125 were independently identified as residues that might be dynamically coupled to each other and correlated to the chemical step [16,24,40,54]; and (iii) Single turnover rates were measured by Benkovic and coworkers for single mutants G121V and M42W and the corresponding double mutant G121V-M42W, which indicated a synergy between the two residues (again, meaning that single mutants caused changes in single turnover rates whose sum was smaller in magnitude than the change generated by the double mutant ) [55].

To investigate the proposition that the above-mentioned residues were part of the dynamic network and hydride tunneling that occurs at the active site, the temperature dependence of the intrinsic KIEs was measured for their single mutants and the corresponding double mutants. Synergism between two such mutants is a direct indicator for coupling between these residues and the chemical step (*i.e.*, the C-H→C hydride transfer per se) [10]. Figure 2 summarizes the isotope effects on the activation energy (ΔE_a_) and the Arrhenius pre-exponential factors (A_H_/A_T_) for the WT DHFR and several of its mutants. The intrinsic KIE studies revealed a temperature-independent KIE of about the same size for the WT and W133F; the data for the other single mutants (M42W, G121V, F125M) exhibited only a modest increase, whereas the double mutants (M41W-G121V, M42W-F125M, G121V-F125M) showed a steep temperature dependence of the KIEs, demonstrating the sizeable influence of these residues on each other and the chemistry. Importantly, the observation that single mutants have a slight effect on the KIE for hydride transfer while the double mutants have a much more dramatic effect accords with the existence of a synergy between those residues suggested by single turnover measurements [43]. Since the effect of the local mutants on the DAD is better understood, Figure 2 also compares the effects of mutation of the active-site residue I14 (green) with the mutation effects of the remote residues. The effect of the single mutation distal to the active site is similar to that of the active site mutation I14V and I14A, while the effect of double distal mutation is similar to that of the active site mutation I14G, indicating in much broader DAD distribution for those double mutants [10].

In summary, coupling between the remote residues affecting the catalyzed chemistry was substantiated for all the experimentally tested residues proposed by QM/MM simulations. However, W133F, a residue that was identified only by bioinformatic studies, did not appear to have an effect on the chemical step. This residue may very well be coupled to other residues affecting protein functions such as folding or solubility, as the bioinformatics study did not focus on the catalyzed chemistry. These experimental investigations confirm the functional role of residues M42, G121, and F125 in the dynamic network of coupled motions correlated to the catalyzed hydride transfer. These outcomes imply that motions of the whole protein provide the environmental reorganization required to maintain an optimum DAD for efficient tunneling.

### 2.3. Heavy DHFR

Study of heavy enzymes, where the normal isotopic distribution of protein atoms is modified, has been shown to be an efficient tool and addresses heated debate regarding the role of protein dynamics in catalysis [19,56,57,58,59,60,61]. Schramm and co-workers initiated the study of heavy enzymes, where the carbons, nitrogens, and non-exchangeable hydrogens (C-H) are substituted to ^13^C, ^15^N, and ^2^H, respectively [56,57]. These enzymes were named “Born-Oppenheimer” enzymes because by making these enzymes heavy, bond vibrations are perturbed with minimal effect on the electrostatics of the protein. Their slower vibrational frequencies could disrupt coordinated vibrations of the protein, and it was proposed that heavy enzymes might be used to assess whether their altered femtosecond-picosecond vibrations affect the catalyzed chemical transformation. The detection of such changes could be instrumental in understanding the relationship between fast protein vibrations and the chemical step, which is one of the main open questions in enzymology today.

**Figure 3:**
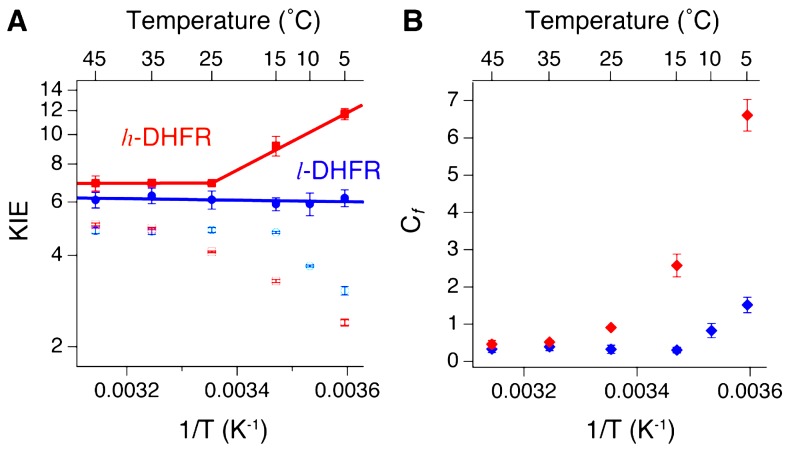
The KIEs and forward commitment factors (*C_f_*) of light DHFR (l-DHFR; blue) and heavy DHFR (*h-*DHFR; red) measured from competitive experiments. (**A**) The observed H/T KIEs (empty symbols) and intrinsic H/T KIEs (filled symbols) are plotted on the logarithmic scale against inverse absolute temperature. The lines are non-linear regression of the intrinsic KIEs to the Arrhenius equation. (**B**) *C_f_* of *h*-DHFR is either equal (25–45 °C) to or larger (5–25 °C) than *C_f_* of *l*-DHFR. Reprinted from ref. [19] with permission from the American Chemical Society.

The analysis of heavy-enzymes is somewhat complicated by the fact that these heavy enzymes are not pure “Born-Oppenheimer” enzymes because some of their electrostatic interactions are also altered. A slight change in geometry and dipole moment in C-H bonds is caused by substituting non-exchangeable hydrogen with deuterium, so aliphatic groups become more hydrophobic while aromatic groups become more hydrophilic [62,63,64,65]. Even though these effects are small, some effects may be multiplied in the extended structure of an enzyme. Therefore, varying protein mass and bond vibrations through isotopic substitution might not only affect the chemical step(s) but also modulate enzyme dynamics on the slower time scales involved in the physical steps of the catalytic cycle (such as binding, conformational changes, and loop movement). For example, kinetic and thermal unfolding experiments have implied that heavy DHFR has altered conformational fluctuation, resulting in protein ligand interactions that differ from light DHFR’s [19]. Measurements of the temperature dependence of intrinsic KIEs of heavy DHFR showed that altered protein mass does not affect the hydride transfer at physiological temperatures (25–45 °C), although a phase transition at 25 °C changes the nature of hydride transfer at lower temperatures (Figure 3). This indicates either a temperature-dependent shift in the protein vibrations coupled to the chemical step or a different conformational ensemble of the heavy enzyme dominating at low temperatures, leading to a broader distribution of DADs for the hydride transfer. Additionally, the forward commitment factors (*C_f_*) on *k*_cat_/*K_M_* in the heavy enzyme are larger, suggesting chemistry is less rate-limiting (Figure 3). The increased *C_f_* of heavy DHFR is likely to be associated with variations in conformational fluctuations that affect the rate of NADPH dissociation from the Michaelis complex. Studies of several other heavy enzymes on different kinetic parameters suggest that the specific dynamics- catalysis relationship may depend on the protein architecture, the nature of the catalyzed reaction, and other physical and chemical properties of the enzymatic system.

## 3. Thymidylate Synthase (TSase)

### 3.1. Introduction

Thymidylate synthase (EC 2.1.1.45, TSase), a pyrimidine-metabolizing enzyme, is one of the most highly-conserved proteins found in nature. This is not surprising as it is involved in the last step of *de novo* synthesis of a precursor of DNA, 2′-deoxythymidine-5′-mono­phosphate (dTMP), using (6*R*)-N^5^,N^10^-methylene-5,6,7,8-tetrahydrofolate (CH_2_H_4_folate) as a cofactor and 2′-deoxyuridine-5′-mono­phosphate (dUMP) as the substrate [66]. The essential role of this enzyme has made it an outstanding target for the development of anticancer therapeutics for several decades. In fact, one of the most commonly used pyrimidine analog drugs in the treatment of cancer is the pro-drug 5-fluorouracil, which, after 5′-phosphorylation in the cells, becomes a covalent inhibitor of TSase. Due to its biological and pharmacological importance, TSase has been studied kinetically and structurally over many years in several theoretical and experimental studies [67,68,69,70].

**Scheme 1:**
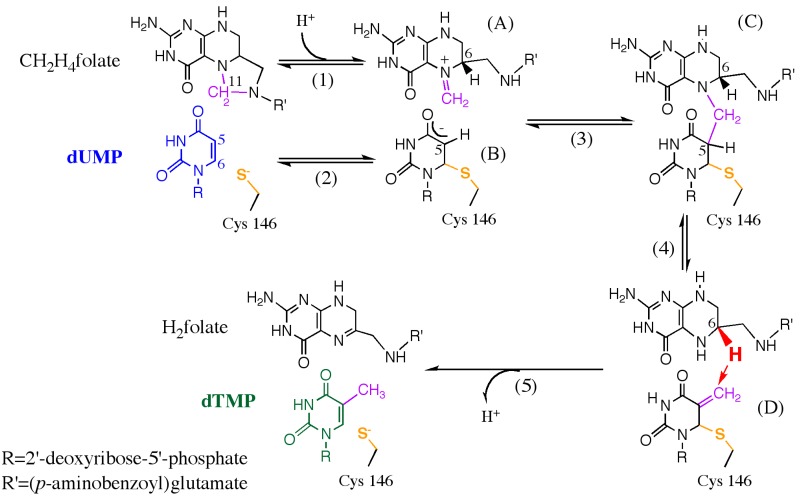
The proposed mechanism for TSase. Adapted from Ref [11] with copyright permission from the American Chemical Society.

The mechanism of TSase (Scheme 1) involves several chemical bond activations including two H transfers: a rate limiting hydride transfer (step 5) and a much faster proton transfer (step 4). Both of these H-transfers have been studied in the WT TSase enzyme via an examination of the temperature dependence of intrinsic KIEs. The intrinsic KIE on the proton transfer is temperature-dependent while that on the hydride transfer is temperature-independent, implying a better-organized transition state (or tunneling ready state) for the latter. QM/MM calculations were used to investigate the molecular details of these experimental observations [71,72,73,74,75]. The calculations confirmed that important protein motions accommodate changes in electrostatic and geometric characteristics of the ligands in both H-transfer reactions.

### 3.2. Y209W TSase

Newby *et al.* used both steady-state kinetics and X-ray crystallography to study the role of a highly conserved residue of TSase from *E. coli* (*ec*TSase), Y209, which is 8 Å away from where the hydride transfer takes place, and contributes one of only two hydrogen bonds to the ribosyl 3′- hydroxyl group of dUMP [76]. These investigators found perfectly superimposable crystal structures of the WT and Y209W ternary complexes with dUMP and CB3717, an analogue of the cofactor (PDB IDs 2G80 and 2G8M, respectively) at a resolution of 1.3 Å. The most conspicuous difference between these two crystal structures was in the anisotropic B-factors, which indicate the direction and amplitude of atomic motions. In contrast to isotropic temperature factors in X-ray diffraction, the ellipsoidal symmetry of the anisotropic B-factors (thermal parameters) provides information regarding the directionality of atomic motions. More explicitly, the orientation and dimension of an ellipsoidal electron distribution is addressed by six anisotropic thermal parameters per atom and indicate amplitudes and directions of atomic vibrations in a given protein crystal. The effect of the mutation on the protein’s vibrational states could be analyzed by comparing the refined anisotropic B-factors of the protein complexes.

**Figure 4:**
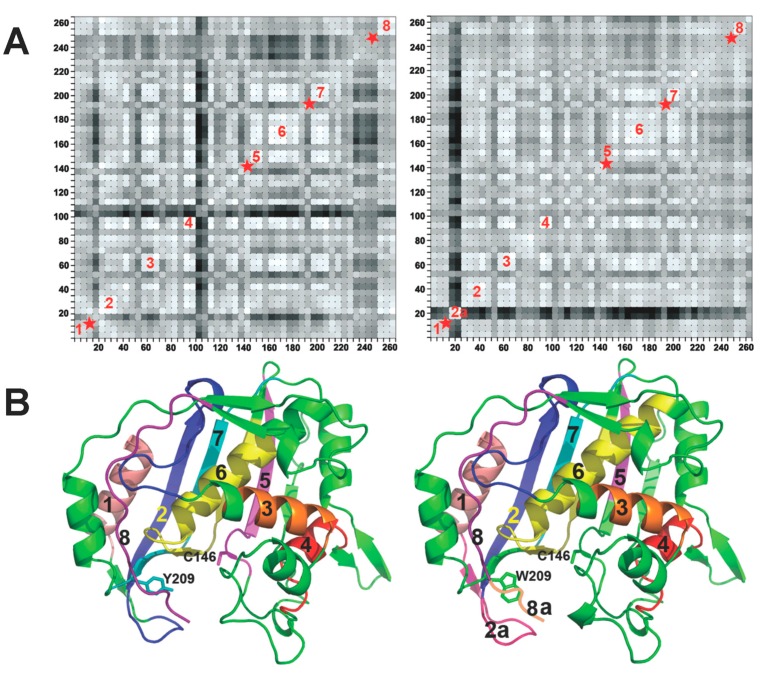
(**A**) The residue-based matrix plots showing correlations of anisotropic B-factor displacements for TSase. The left plot is for WT TSase and the right plot is for Y209W TSase. Segment 2a in the right plot is the phosphate-binding loop with relatively higher B factors. (**B**) Ribbon diagrams of WT (left) and Y209W (right). The mutated residue, dUMP, catalytic cysteine and cofactor analog (CB3717) are shown as sticks. Reproduced from ref [11] with permission from the American Chemical Society.

The correlation in movement of any two atoms can be measured by the differences in the projections of their anisotropic thermal parameters along their interatomic vector. In the matrix plot (Figure 4A), the degree of correlation of atoms is represented by color-coding, where the lighter shades of grey indicate greater correlation. The blocks of light-colored squares in the plots indicate the protein residues that vibrate as rigid bodies. The refined anisotropic B-factors of the crystal structures revealed that some protein segments of Y209W have disrupted rigid-body vibrations compared to WT TSase’s (Figure 4A). The anisotropic B-factors of atoms in several loops across the WT protein are all oriented in the same direction suggesting a concerted movement of these loops in the WT TSase. However, in Y209W, the anisotropic B factors of those segments are randomly oriented, indicating a disruption of the correlated atomic vibrations of protein residues in the mutant and these are marked by red stars in Figure 4.

The examination of the structural and kinetic properties of this mutant was extended, and compared to the temperature dependence of KIEs on the WT- and Y209W-catalyzed hydride transfer reactions [11]. The correlation between the structural and kinetic data allows a distinction to be made between mutational effects on protein motions that influence the hydride transfer reaction and protein motions that influence other catalytic steps. For example, the substitution of tyrosine to tryptophan dramatically affected the *K_M_* values for the substrate and cofactor. Interestingly, the trend was more pronounced for the cofactor of the enzyme, CH_2_H_4_folate, although its binding site is even more remote from the mutation’s site than is the substrate’s. The higher *K_M_* for CH_2_H_4_folate in Y209W indicates a need to modulate contacts between different segments of the protein.

The higher *K_M_* of CH_2_H_4_folate in Y209W is in accordance with the conformation of position 209 prior to hydride transfer, being more disordered and further away from dUMP than in the WT. This is in agreement with the significant changes in the average B factors of several loops in the Y209W crystal structure compared to the WT. Greater mobility of these loops could impair the binding of the substrate and cofactor. Subsequently, the effect of the Y209W mutation on the mobility of this loop can propagate to the other regions in the active site cavity.

Additionally, it was found that in the Y209W mutant, the intermediate that is formed prior to hydride transfer creates a thiol-trapped by-product [11]. This finding indicated that the mutation no longer forced the H-donor and acceptor close together, allowing a small thiol to compete for intermediate D (Scheme 1). Furthermore, the altered dynamics of the H-donor and acceptor were probed by measuring the temperature dependence of the intrinsic KIEs of Y209W, which indicated a disrupted distribution of DAD. Inflated temperature dependence of the KIEs were found in Y209W [11], which reflects alteration of vibrations at the picosecond-femtosecond timescale: the sampling of DADs at the reactive state is commonly predicted to be at frequencies as low as 50–200 cm^−1^ (~0.5 picosecond) [50,77,78,79,80]. The larger temperature dependence in the Y209W mutant suggests a less perfect reorganization for hydride transfer. The findings suggested that this dynamically altered mutant partly distorts the well-defined reactive state for hydride transfer in the WT enzyme [11].

### 3.3. Effect of Mg^2+^ on Motions of WT TSase

Although Mg^2+^ has been reported to affect TSase activity [81,82], the mechanism has not been investigated until recently [83]. In order to correlate the kinetic effects of Mg^2+^ with their structural and dynamic origins, two X-ray crystallographic structures (PDB IDs 2G80, 4IW5 and 4ISK) were re-analyzed. Due to the differences in the stereochemistry of each inhibitor’s glutamyl moiety, the possible Mg^2+^ sites in the ternary complex of TSase differ in these crystal structures. However, in both cases the cation, Mg^2+^ was identified near the glutamyl moiety of the folate cofactor of TSase, *ca.* 16 Å away from the active site [83].

**Figure 5:**
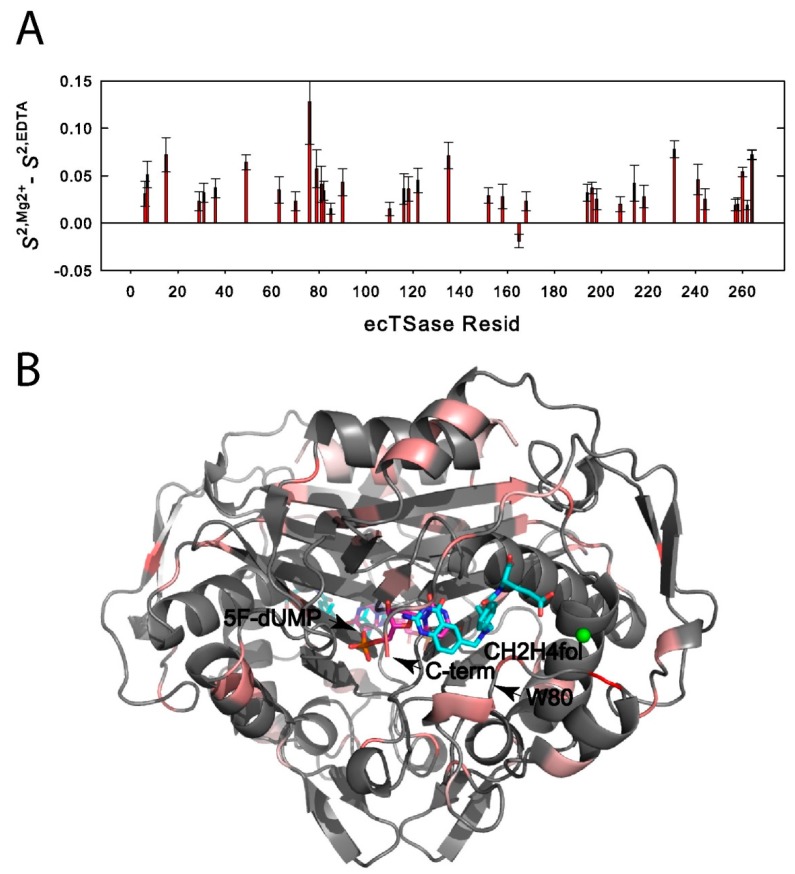
(**A**) Mg^2+^ binding rigidifies the TSase-(5F-dUMP)-CH_2_H_4_fol complex. Significant (greater than 2σ) differences in ^15^N-^1^H order parameters (Δ*S*^2^ = *S*^2, Mg2+^-*S*^2, EDTA^) are shown. (**B**) The significant changes in panel A are highlighted on a ternary complex structure with a color gradient of white to red representing minimal to maximal changes in *S*^2^. Residues with no significant change are colored in and the suggested binding site for Mg^2+^ is shown in green. Reproduced from ref [83] with permission from the American Chemical Society.

NMR relaxation experiments were used to investigate the effects of Mg^2+^ on the protein dynamics. These employed the generalized order parameters (*S*^2^), which signifies the rigidity of the protein, and ranges from complete disorder (*S*^2^ = 0) to fixed bond orientation (*S*^2^ = 1) on the picosecond-nanosecond timescale. The *S*^2^ for backbone ^15^N-^1^H vectors in the TSase-(5F-dUMP)-CH_2_H_4_fol complex was measured with and without Mg^2+^. Significant differences in *S*^2^ upon Mg^2+^ binding was observed, which is consistent with a model in which Mg^2+^ binding lowers the entropic barrier of the hydride transfer by paying some of the conformational entropy penalty in the ground state. In other words, the Mg^2+^-induced rigidification of the enzyme leads to a larger fraction of reactive conformers. Moreover, Mg^2+^ binding rigidifies regions of TSase across the protein, and stabilizes the closed conformation (*i.e.*, the loop containing W80 and W83 and the C-terminus). These dynamic effects extend beyond the metal binding region, as distal bond vectors, including some at the dimer interface (I29, G31, F36, F152, K158, Q165, and D198) show significant changes (Figure 5).

## 4. Conclusions

The role of protein motions in enzyme catalyzed bond activation has been extensively studied for many years. The experiments described above illuminate aspects of how protein motions affect different H-transfer steps in different reaction mechanisms, and extend our understanding of enzyme catalysis. Those studies used a combination of different experimental techniques, including temperature dependence of intrinsic KIEs, steady state kinetics, site-directed mutagenesis, NMR and X-ray crystallography to study two different model enzymatic systems, TSase and DHFR.

One of the contemporary advances in enzymology is the use of KIEs in exploring the role of motions in enzymes’ function. The relevance of temperature dependence of KIEs to the enzyme’s dynamics was put forward in 1999 [84], and has since played a pivotal role in enzymology. In this review, the temperature dependence of intrinsic KIEs has been applied to reveal various aspects of the hydrogen transfer reactions catalyzed by the two enzymes, DHFR and TSase. The findings presented above provide another piece of the grand puzzle describing the relationship between motions and chemical reactivity in enzymes. In addition to their merit for basic-science, such investigations may provide new insights for rational drug designs that target these, and other enzymes. Finally, an understanding of the relationship between enzyme function, structure, and dynamics in covalent bond activation could in the long run enable a better *de novo* design of artificial biocatalysts and will substantially enhance our knowledge and capability to control enzyme catalysis.
